# Early warning value of multiple serum indicators combined with ultrasound detection in girls with central precocious puberty

**DOI:** 10.3389/fendo.2025.1518764

**Published:** 2025-04-07

**Authors:** Xiaolong Song, Jianping Zhou, Tiantian Han, Zhifeng Lin, Xu Chen, Yufang Li

**Affiliations:** ^1^ Radioimmunoassay Center, Shaanxi Provincial People’s Hospital, Xi’an, Shaanxi, China; ^2^ Pediatric Internal Medicine Department, Shaanxi Provincial People’s Hospital, Xi’an, Shaanxi, China; ^3^ Rehabilitation Center of Joint Logistic Force of PLA, Dalian, Liaoning, China; ^4^ State Key Laboratory of Cancer Biology, Department of Biochemistry and Molecular Biology, Fourth Military Medical University, Xi’an, Shaanxi, China; ^5^ Institute of Medical Research, Northwestern Polytechnical University, Xi’an, Shaanxi, China

**Keywords:** precocious puberty, premature thelarche, basal LH, IGF-1, IGFBP-3, DHEAS

## Abstract

**Aim:**

The aim of this study was to evaluate the early diagnostic potential of various serum biomarkers and ultrasound characteristics in girls diagnosed with early central precocious puberty (CPP).

**Methods:**

A cohort of 125 girls presenting breast development was assessed between May 2020 and January 2023. Following a six-month follow-up and GnRH agonist stimulation test, 78 girls were classified into the CPP group and 47 into the premature thelarche (PT) group. Serum biomarkers, including insulin-like growth factor-binding protein 3 (IGFBP-3), insulin-like growth factor 1 (IGF-1), and dehydroepiandrosterone sulfate (DHEAs), as well as bone age index (BAI) and ultrasound features, were compared between the groups.

**Results:**

Chronological age did not significantly differ between the groups, but bone age and BAI were notably higher in the CPP group. Most serum levels, except for peak FSH, were significantly elevated in the CPP group. Ultrasound metrics such as uterine volume, cervical ratio, ovarian volume, and milk bud characteristics also showed significant differences. Correlation analyses revealed positive associations between both physical and serum indices and peak LH levels. Receiver operating characteristic (ROC) curve analysis identified basal LH as having the highest area under the curve (AUC) of 0.927, followed by DHEAs (AUC = 0.924). Logistic regression identified LH and DHEAs as independent risk factors for CPP, with optimal diagnostic efficacy achieved when both markers were combined (AUC = 0.973).

**Conclusion:**

Basal LH, IGF-1, IGFBP-3, DHEAs, and associated ultrasound features are valuable early indicators for CPP in girls. The combination of LH and DHEAs enhances diagnostic accuracy, establishing them as significant independent risk factors for CPP.

## 1 Introduction

The term “precocious puberty” describes secondary sexual development that begins before the age of eight. Based on the activation of the hypothalamic-pituitary-gonadal axis (HPGA), it is categorized into incomplete precocious puberty, peripheral precocious puberty, and central precocious puberty (1). Central precocious puberty (CPP), also known as GnRH-dependent precocious puberty, originates from increased secretion of gonadotropin-releasing hormone (GnRH) by the hypothalamus, leading to premature activation of the gonadal axis and subsequent development of secondary sexual characteristics. Girls with CPP typically exhibit accelerated growth, breast development, advanced bone age, and rapid epiphyseal closure, often resulting in short stature in adulthood (2). Premature thelarche (PT) is a form of incomplete precocious puberty characterized solely by breast development without other signs of sexual maturation and generally resolves spontaneously (3). Early onset of secondary sexual characteristics can result in negative psychological outcomes, including anxiety and low self-esteem. In addition, it may lead to undesirable health outcomes in adulthood, such as depression and substance abuse. For this reason, a timely and accurate diagnosis of CPP is essential.

The GnRH stimulation test is currently the gold standard for identifying HPGA activation (4), but it is time-consuming, with poor compliance and potential side effects. The European guidelines for GnRHa application recommend limited use of the GnRH stimulation test (5). Consequently, simpler and more effective early diagnostic indicators are urgently needed. Recent studies have indicated that the acceleration of growth in adolescence is closely related to the GH-IGF-1 axis (6). IGFBP-3, which binds with high affinity to insulin-like growth factor (IGF), reflects gonadotropin secretion levels but its diagnostic value as a biomarker for precocious puberty remains uncertain (7).

Recent findings suggest that basal morning blood levels of GnRH can serve as indicators for CPP (8). Alterations in baseline blood LH, the rate of gonadal and sexual development, height, and the growth velocity change are key parameters for monitoring CPP progression (9), though their utility as early diagnostic indicators in clinical practice remains uncertain. DHEAs, reflecting adrenal androgen content and activity, are identified as a high-risk factor for early menarche and breast development in girls (10), supporting its potential role in studying gonadal development. However, the clinical utility of these factors in early CPP diagnosis remains to be fully elucidated.

The aim of this study was to evaluate the diagnostic and differential diagnostic value of CPP in girls, providing a reference for clinical diagnosis. The study analyzes the quantification of GnRH through morning blood tests as an indicator for CPP, along with alterations in LH levels, the rate of gonadal and sexual development, height, and the growth velocity change as key parameters for monitoring CPP progression.

## 2 Materials and methods

### 2.1 Inclusion and exclusion criteria

A total of 125 girls diagnosed with breast development were recruited from May 2020 to January 2023 at Shaanxi Provincial People’s Hospital. The study received approval from the Medical Ethics Committee of Shaanxi Provincial People’s Hospital (No.: 2021105) and was conducted in accordance with the Declaration of Helsinki.

Inclusion criteria for CPP (N = 78):

1. Secondary sexual development before the age of eight.
2. Accelerated linear growth: annual growth rate significantly higher than normal (more than 2 standard deviations above the mean).
3. Advanced bone age: bone age at least one year older than chronological age.
4. Gonadal enlargement: increased ovarian and uterine size with several follicles (diameter > 4 mm) detected via pelvic ultrasound.
5. Initiation of HPGA: serum gonadotropin and sex hormone levels reaching puberty thresholds (GnRH stimulation tests: peak LH levels > 5 IU/L and/or peak LH/FSH ratio > 0.6) (1).

Inclusion criteria for PT (N = 47):

1. Breast development before the age of eight.
2. Absence of other signs of sexual development.
3. No growth acceleration or advanced skeletal development.
4. Absence of vaginal bleeding.

Exclusion criteria:

1. Secondary sexual characteristics originating from central nervous system abnormalities, congenital adrenal hyperplasia (CAH), congenital hypothyroidism, or exogenous drugs.
2. Malnutrition and other urogenital diseases affecting menarche and growth.
3. Insufficiency of critical organs such as the heart, liver, and kidneys.

### 2.2 General clinical data

All subjects were evaluated by a single pediatric endocrinologist, using criteria such as Tanner stage, height, and weight. Height was measured to the nearest 0.1 cm and weight to the nearest 0.1 kg. Body mass index (BMI) was calculated using the formula: BMI = weight/height². Bone age was assessed using the Greulich-Pyle method, and the Bone Age Index (BAI) was calculated as BAI = bone age (BA)/chronological age (CA). Pelvic and breast ultrasound features included uterine volume (upper and lower diameter × anterior and posterior diameter × transverse diameter × 0.5233), ovarian volume (selected data from dominant side), follicle number (diameter ≥ 4 mm), milk bud diameter, and milk bud volume (longitudinal diameter × anterior and posterior diameter × transverse diameter × 0.5233).

### 2.3 Serum sample collection and detection

Fasting venous blood samples (3 mL) were collected between 8:00 and 9:00 am, centrifuged at 4°C, 2,500 g, and stored at -80°C until analysis. Serum IGF-1 and IGFBP-3 levels were measured using the Immulite 2000 Chemiluminescence Immunoassay Kit (SIEMENS Healthcare Diagnostics Products Limited, United Kingdom). The intra- and inter-assay coefficients of variation (CVs) provided by the manufacturer were < 2.4% and < 4.7%, and < 4.8% and < 5.2%, respectively. Detection limits for IGF-1 and IGFBP3 were 1,600 ng/mL and 16 µg/mL, respectively. Serum LH, FSH, and estradiol (E2) levels were measured using direct chemiluminescence (SIEMENS Healthcare Diagnostics Products Limited, United Kingdom), with CVs reported as < 2.6% and < 3.7%, < 3.2% and < 5.3%, and < 2.6% and < 0.9%, respectively. Detection limits for LH, FSH, and E2 were 200 IU/L, 200 IU/L, and 3,000 pg/mL, respectively.

### 2.4 GnRH stimulation test

The GnRH stimulation test was conducted in the early morning. Subjects, who were awake and fasting, received an injection of 2.5 μg/kg gonadorelin (maximum dose of 100 µg). Blood samples were collected at 0, 30, 60, 90, and 120 minutes post-injection to measure serum FSH and LH levels. A stimulated LH level > 5 IU/L or a peak LH to peak FSH ratio (LH/FSH) > 0.6 was considered indicative of HPGA activation (1).

### 2.5 Statistical analysis

Statistical analysis was performed using SPSS 25.0 software. The means ± standard deviation (SD) of normally distributed measurement data were presented, and the t-test was used for comparison. Mann-Whitney U analysis was used to examine non-normally distributed data, which were presented as medians. The Pearson correlation test was used for group-to-group correlation analysis. The diagnostic viability of the indicators under investigation was assessed using multivariate logistic regression and receiver operating characteristic (ROC) curve analysis. Differences were considered statistically significant at p < 0.05.

## 3 Results

### 3.1 Clinical, biochemical and ultrasound characteristics in CPP and PT

A total of 78 girls with CPP (7.83 ± 0.72 years old) and 47 girls with PT (7.77 ± 0.96 years old) were enrolled in this study. General characteristics of the two groups are shown in **Table 1**. There was no significant difference in chronological age (CA) between CPP and PT groups (p = 0.419). However, bone age (BA) and bone age index (BAI) were higher in the CPP group, with significant differences (p = 0.001, p < 0.001). Basal LH, FSH, IGF-1, IGFBP-3, and DHEAs levels were significantly different between the CPP and PT groups (p < 0.01), except for the peak FSH level (p = 0.046). Ultrasound indices, including uterine volume, cervical ratio, ovarian volume, follicle number (≥4 mm), milk bud diameter, and milk bud volume, also showed significant differences between the two groups (p < 0.001).

**Table 1:** Clinical and biochemical characteristics of the subjects.

	CPP (n=78)	PT (n=47)	P-Value	T
CA (year)	7.84 ± 0.72	7.71 ± 0.88	0.419	0.812
BA (year)	9.27 ± 1.19	8.53 ± 1.22	0.001	3.318
BAI (BA/CA)	1.18 ± 0.08	1.10 ± 0.08	<0.001	5.041
Height (cm)	138.69 ± 12.96	131.56 ± 10.59	0.001	3.341
Weight (kg)	32.23 ± 8.05	29.22 ± 8.01	0.045	2.022
BMI (kg/cm^2^)	16.59 ± 2.73	16.57 ± 2.58	0.978	0.028
Uterine volume (mL)	2.23 ± 1.31	1.34 ± 0.69	<0.001	4.297
Cervical ratio	1.24 ± 0.22	1.04 ± 0.09	<0.001	5.953
Ovarian volume (mL)	2.25 ± 1.08	1.30 ± 0.66	<0.001	5.867
Follicle number (≥4mm)	3 (2,4)	1 (1,2)	<0.001	5.465
Milk bud diameter (cm)	0.96 ± 0.37	0.66 ± 0.33	<0.001	4.791
Milk bud volume (cm^3^)	0.31 ± 0.18	0.15 ± 0.11	<0.001	5.565
E_2,_ (pg/ml)	15.03 ± 6.38	9.90 ± 1.31	<0.001	5.438
Basal LH (IU/L)	0.93 ± 0.49	0.29 ± 0.12	<0.001	9.097
Basal FSH (IU/L)	4.43 ± 2.29	2.41 ± 1.22	<0.001	5.559
LH/FSH	0.24 ± 0.15	0.13 ± 0.06	<0.001	4.613
Peak LH (IU/L)	17.43 ± 13.61	4.96 ± 1.14	<0.001	6.252
Peak FSH (IU/L)	17.42 ± 6.42	15.34 ± 5.00	0.046	2.020
LH/FSH (Peak)	1.01 ± 0.81	0.34 ± 0.09	<0.001	5.847
IGF-1 (ng/mL)	314.70 ± 110.19	194.62 ± 45.83	<0.001	7.100
IGFPB-3 (ug/mL)	6.45 ± 0.87	4.50 ± 1.12	<0.001	10.595
DHEAs (ng/mL)	103.68 ± 31.06	55.78 ± 21.02	<0.001	9.348

CPP, central precocious puberty; PT, premature thelarche; n, number; CA, chronological age; BA, bone age; BMI, body mass index; BAI, bone age index.

### 3.2 Correlation analysis of predicting indicators

Spearman correlation analysis (**Table 2**) revealed positive associations between peak LH and LH/FSH (peak) with both physical and serological indicators (p < 0.001). Among ultrasound characteristics, cervical ratio and ovarian volume were positively correlated with peak LH and LH/FSH (peak) (p < 0.01). Milk bud diameter and volume were positively correlated with LH/FSH (peak) (p < 0.01).

**Table 2:** correlation analysis between all predictive markers with peak LH and peak LH/FSH.

	Peak LH		LH/FSH (Peak)	
	r	p-Value	r	p-Value
BA (year)	0.487	<0.001	0.446	<0.001
BAI (BA/CA)	0.403	<0.001	0.380	<0.001
Height (cm)	0.318	<0.001	0.355	<0.001
Weight (kg)	0.425	<0.001	0.450	<0.001
BMI (kg/cm^2^)	0.335	<0.001	0.327	<0.001
Uterine volume (mL)	0.071	0.433	0.025	0.779
Cervical ratio	0.251	0.005	0.233	0.009
Ovarian volume (mL)	0.375	<0.001	0.326	<0.001
Follicle number (≥4mm)	0.188	0.036	0.141	0.117
Milk bud diameter (cm)	0.177	0.048	0.304	0.001
Milk bud volume (cm^3^)	0.160	0.046	0.276	0.002
E_2_ (pg/ml)	0.514	<0.001	0.537	<0.001
Basal LH (IU/L)	0.669	<0.001	0.601	<0.001
Basal FSH (IU/L)	0.570	<0.001	0.426	<0.001
IGF-1 (ng/mL)	0.486	<0.001	0.441	<0.001
IGFPB-3 (ug/mL)	0.333	<0.001	0.328	<0.001
DHEAs (ng/mL)	0.664	<0.001	0.747	<0.001

### 3.3 The ROC curve results of all predicting indicators separated

ROC curve analysis was performed to determine the optimal cut-off values for distinguishing CPP from PT for each indicator based on sensitivity, specificity, and area under the curve (AUC). Basal LH had the highest AUC (0.927), with a cut-off value of 0.47 IU/L (sensitivity 91.0%, specificity 85.1%), followed by DHEAs (AUC = 0.924, cut-off 77.83 ng/mL, sensitivity 96.2%, specificity 83.0%).

### 3.4 Multivariate logistic regression analysis of the CPP early warning indicators

**Figure 1:**
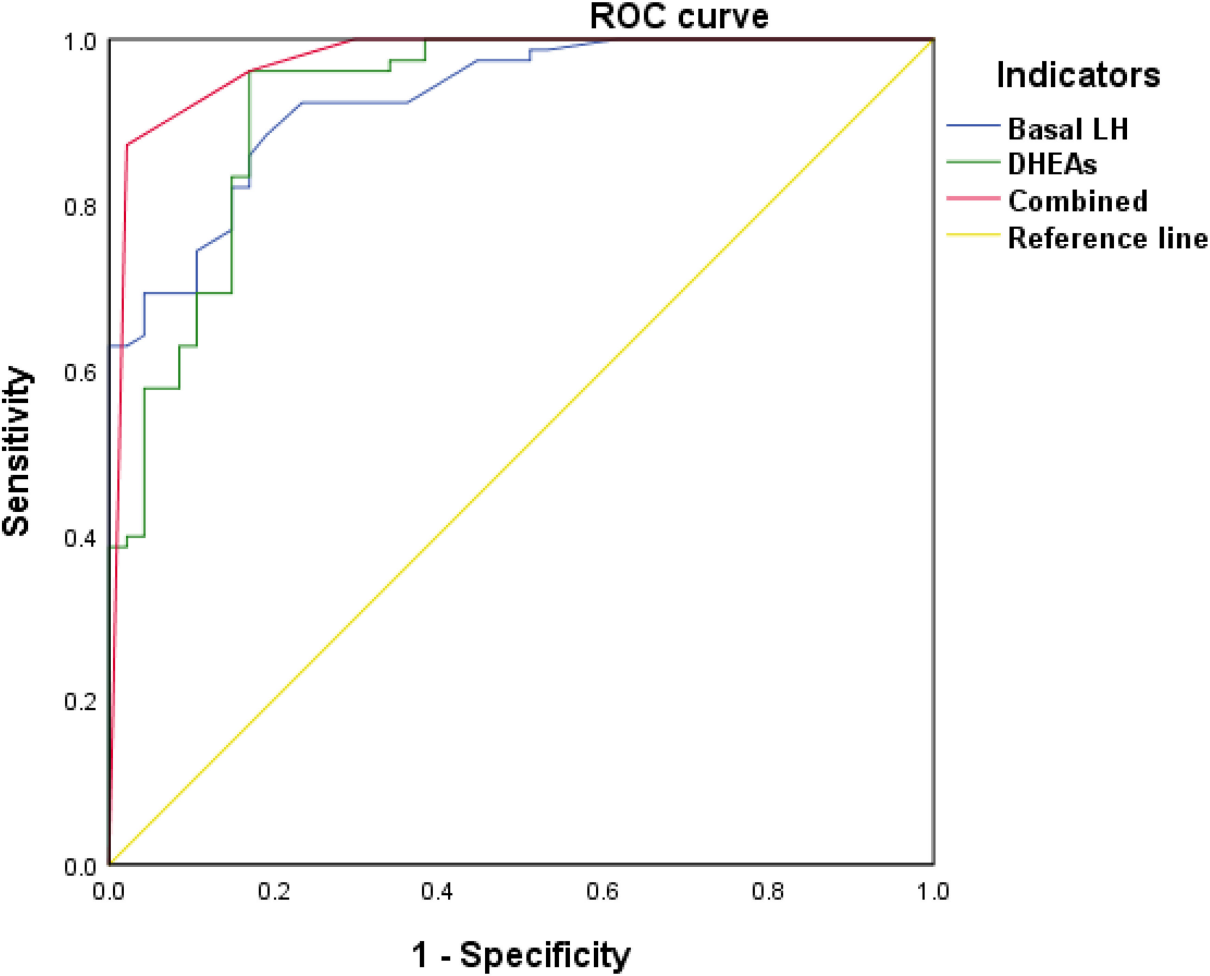
Results of ROC curves for LH, DHEAs separated and combined.

Following univariate analysis, variables listed in **Table 3** were included as independent variables (values greater than the cut-off were assigned “1”), and CPP was treated as the dependent variable (CPP assigned “1” and PT assigned “2”). Logistic regression analysis (**Table 4**) identified LH and DHEAs as risk factors for CPP. The combined test showed the highest predictive efficacy for CPP (AUC: 0.973, 95% CI = 0.944-1.000, p < 0.001) (**Figure 1**).

**Table 3:** The ROC curve results of all predicting indicators separated.

	AUC	P-Value	95%CI	Cut-off	Youden	Sensitivity	Specificity
Uterine volume (mL)	0.719	<0.001	0.629-0.809	1.26	0.416	75.6	66.0
Cervical ratio	0.824	<0.001	0.752-0.897	1.11	0.509	61.5	89.4
Ovarian volume (mL)	0.773	<0.001	0.689-0.857	1.61	0.501	75.6	74.5
Follicle number (≥4mm)	0.786	<0.001	0.707-0.864	1.5	0.417	82.1	59.6
Milk bud diameter (cm)	0.755	<0.001	0.667-0.843	0.65	0.407	83.3	57.4
Milk bud volume (cm^3^)	0.783	<0.001	0.701-0.865	0.16	0.458	75.6	70.2
E_2_ (pg/ml)	0.879	<0.001	0.818-0.940	10.32	0.698	83.3	87.2
Basal LH (IU/L)	0.927	<0.001	0.895-0.976	0.47	0.761	91.0	85.1
Basal FSH (IU/L)	0.797	<0.001	0.718-0.875	3.08	0.505	71.8	79.7
IGF-1 (ng/mL)	0.866	<0.001	0.803-0.930	232.72	0.667	79.5	81.2
IGFPB-3 (ug/mL)	0.903	<0.001	0.849-0.957	4.98	0.702	93.6	70.2
DHEAs (ng/mL)	0.924	<0.001	0.846-0.963	77.83	0.792	96.2	83.0

**Table 4:** Multivariate Logistic regression analysis of the CPP early warning indicators.

	B	SE	Wald	P	OR	95%CI
LH	4.462	1.104	16.336	<0.001	86.667	9.958-754.326
DHEAs	5.194	1.163	19.935	<0.001	180.134	18.427-1760.940
Constant	-14.899	3.266	20.809	<0.001		

## 4 Discussion

Adolescence is a critical period characterized by growth in height and weight, reproductive organ development, and increased neuroendocrine activity (11). CPP results from the premature onset of puberty, with a higher incidence in girls than boys. The early development associated with CPP can lead to significant psychosocial issues, making timely diagnosis and intervention vital (12). While variations in breast development are often noticeable, most children are diagnosed after the age of eight. Typically, uterine and ovarian enlargement occurs after nine years of age, and early breast development can be a common feature of both CPP and PT, complicating early differential diagnosis.

Currently, the GnRH stimulation test is the standard method for CPP diagnosis, although it has limitations, including the potential for false negatives. This necessitates a comprehensive analysis of secondary sexual development, growth patterns, and bone age (13). Bone age (BA) reflects biological age and is a key indicator of abnormal growth. BAI provides a more accurate representation of bone maturation in children of different ages. In this study, height, weight, BA, and BAI were all higher in the CPP group compared to the PT group, indicating increased growth velocity in CPP children.

Ultrasound, being non-invasive and reproducible, offers valuable insights into breast, uterus, and ovarian development. However, the overlap in ovarian and uterine volumes between prepubertal and early pubertal girls can complicate differentiation. This study included milk bud diameter and volume to evaluate breast development, minimizing the impact of factors such as body fat and dietary habits. The significant differences in ultrasound indices between CPP and PT groups demonstrate the utility of ultrasound in differential diagnosis.

A complex hormonal cascade including E2, LH, FSH, and IGF-1, is involved in pubertal development. Basal LH is particularly important for identifying HPGA activation (14–16). Due to the early initiation of the HPGA axis, children with CPP exhibit higher levels of LH, which can be better represented by morning blood samples (17). Recent guidelines suggest reducing the use of GnRH/GnRHa stimulation tests, favoring basal LH as the best biochemical index for CPP diagnosis (5). However, the Chinese consensus highlights limitations due to the pulsatile secretion of LH and its sensitivity to BMI and detection methods (18). Our data showed significant increases in basal E2, LH, and FSH in the CPP group compared to the PT group. Furthermore, basal LH was strongly correlated with peak LH and LH/FSH (peak), supporting its role in HPGA activation (19). ROC curve analysis confirmed that basal LH is a highly effective diagnostic marker for CPP, with an AUC of 0.927 and a cut-off value of 0.47 IU/L, providing 91.0% sensitivity and 85.1% specificity.

IGF-1 plays a critical role in child growth, particularly in promoting linear growth. IGF-1 is involved in cell proliferation, differentiation, maturation, and metabolic balance, and its synthesis increases with HPGA activation (20). IGFBP-3, with its high affinity for IGF, reflects gonadotropin secretion levels, indicating growth and sex axis development (21). DHEAs, which are related to adrenal androgens, influence breast development and gonadal maturation, potentially affecting height (22). In this study, basal levels of IGF-1, IGFBP-3, and DHEAs were significantly elevated in the CPP group. DHEAs correlated with LH peak and LH/FSH (peak), indicating its role in early puberty initiation. In evaluating children with signs of early sexual maturation, it is essential to consider premature adrenarche, characterized by the early appearance of pubic hair and elevated DHEA-S levels, often accompanied by advanced bone age but without breast development. This condition can mimic aspects of central precocious puberty, complicating the differential diagnosis. While DHEA-S is a useful marker for both conditions, its elevation alone does not suffice for a CPP diagnosis. Therefore, a comprehensive assessment of clinical indicators is necessary to differentiate between CPP and premature adrenarche.

The ROC curve analysis demonstrated that DHEAs had a high AUC of 0.924, indicating excellent diagnostic performance in differentiating CPP from PT. With a cut-off value of 77.83 ng/mL, DHEAs exhibited a sensitivity of 96.2% and specificity of 83.0%, making it a highly reliable marker for clinical use. This superior performance compared to IGFBP-3 (AUC 0.903) and IGF-1 (AUC 0.866) highlights DHEAs’ potential as an auxiliary diagnostic tool. Its strong correlation with LH peak and LH/FSH ratios further supports its relevance in assessing hypothalamic-pituitary-gonadal axis activation. Integrating DHEAs measurement into routine diagnostics could enhance accuracy, reduce reliance on more invasive tests, and ultimately improve early intervention strategies for children with CPP.

The combined analysis of basal LH and DHEAs, identified as independent CPP risk factors through logistic regression, significantly improves diagnostic efficacy. With an AUC of 0.973, this combined approach offers a reliable, cost-effective, and less invasive diagnostic method suitable for early CPP detection. However, this single-center study has limitations. Future studies should involve multi-center studies to validate the differential expression of basal LH and DHEAs in children with CPP across different regions. Long-term follow-up is also necessary to monitor the progression of puberty and the changes in basal LH and DHEAs in children with varying CPP progression rates.

## 5 Conclusion

This study indicates the significant differences between central precocious puberty (CPP) and premature thelarche (PT) in growth parameters, hormonal profiles, and ultrasound findings. The early onset of puberty, particularly in CPP, requires timely identification and intervention due to the associated psychosocial challenges. Our findings demonstrate that basal LH, along with other hormonal markers such as IGF-1, IGFBP-3, and DHEAs, are robust indicators for diagnosing CPP, potentially reducing the need for invasive stimulation tests. In addition, the use of ultrasound to assess breast and pelvic development provides a non-invasive approach to differentiate between CPP and PT more effectively. These insights highlight the importance of a comprehensive evaluation that includes clinical, hormonal, and imaging assessments to enhance diagnostic accuracy and clinical management of early puberty in girls.

## Data Availability

The original contributions presented in the study are included in the article/supplementary material. Further inquiries can be directed to the corresponding authors.
